# Current situation of pediatric clinical trials in China: focus on trials for drug marketing application and administrative approval

**DOI:** 10.1186/s12887-022-03208-2

**Published:** 2022-03-18

**Authors:** Lin Song, Yuntao Jia, Sujuan Ran, Bin Li, Jin Xu, Bennian Huo, Nange Yin, Maolin Ai, Yao Liu

**Affiliations:** 1 Department of Pharmacy, National Clinical Research Center for Child Health and Disorders, Ministry of Education Key Laboratory of Child Development and Disorders, China International Science and Technology Cooperation base of Child development and Critical Disorders, Children’s Hospital of Chongqing Medical University, Chongqing, 400014 China; 2 Department of Pharmacy, Daping Hospital, Army Medical University, Chongqing, 400042 China

**Keywords:** Pediatric patients, Clinical trials, Drug, Dosage form, Drug Trial Registration and Information Publication Platform

## Abstract

**Background:**

Research and development of pediatric drug faces many difficulties and pediatric clinical trials remain a challenge. Since 2011, a series of measures have been taken to encourage research, development of drugs for pediatric patients in China. In this study, we analyzed pediatric clinical trials conducted in China to provide reference for research and development of pediatric drugs and formulation of relevant policies.

**Methods:**

We conducted a cross-sectional observational study of pediatric trials registered in the Drug Trial Registration and Information Publication Platform before Oct. 31, 2021. All trials that recruited children (under 18 years old as defined in China) were retrieved and general characteristics of the trials and the research drugs were extracted and analyzed. The data were extracted and statistically analyzed by excel 2010 and SPSS 22.0, respectively.

**Results:**

There were 588 registered pediatric clinical trials, which accounted for 3.94% of the total registered trials. The overall average annual growth rate of the number of trials from 2013 to 2020 was 14.47% (*P* < 0.01). Of the 588 trials included, there were 312 trials (53.06%) with only children as subjects, 127 trials (21.60%) with research drugs only for children use, and the median of target subject number was 320 with the range of 8 to 600,000. The sponsors and the principal investigators were mainly located in the eastern and northern China. 325 trials were vaccine trials, and the dosage form was mainly injection. There were 98 non-vaccine biological product trials (mainly injections), 135 chemical compound drug trials (mainly tablets), 30 traditional Chinese medicine/natural drugs (mainly granules). Indications of the non-vaccine drugs were mainly diseases of the blood and blood-forming organs and certain disorders involving the immune mechanism.

**Conclusion:**

The number of pediatric clinical trials in China has increased these years. To further promote pediatric clinical trials and motivate pediatric appropriate drug marketing application and administrative approval, conducting large pediatric clinical trials, further development of dosage forms suitable for children with special attention to neonates and prematurity, and improving uneven geographical distribution of sponsors and researchers are the current challenges.

## Background

Research and development of pediatric drug faces many difficulties, such as ethical issues, difficult to carry out informed consent effectively. Besides, as neonates, infants, children, and adolescents are in different physiological development stage, a drug may need to be studied separately in those groups, then, the complexity and high cost both lead to a lack of pediatric drug research [1]. Previously studies showed the approval process for a new drug in the United States took an average of 12 years but approval using in pediatrics need a lag time of another 8 ~ 10 years [2], and the lag time was about 1017 days in Japan and the European Union [3]. Although legislative actions have clearly defined and provide incentives for appropriate research on children in some countries, such as United States and the European Union [4, 5], to stimulate the development of pediatric drugs and provide more information on their use, recent reports on the effects of legislation indicated that pediatric clinical trials remained a challenge [6]. Due to lack of specific drugs and clinical trial results, off-label use of drugs is widespread in children. Previous studies showed the incidence of off-label medication use ranged from 28.3 to 46.5% in pediatrics [7–10], and it was associated with occurrence of adverse drug events [11, 12], posing a great hidden danger to medication therapy and patient safety [13]. Therefore, strategies and initiatives to promote pediatric clinical trials always has been concerned all over the world.

Since 2011, a series of measures have been taken to encourage the research, development and production of specific drugs and their suitable dosage forms for pediatric patients in China [13]. In May 2014, the former State Health and Family Planning Commission and other six ministries and commissions have issued “Several Opinions on Safeguarding Drug Use for Children” (No.29 [2014] of the State Health and Family Planning Commission) [14], setting forth specific requirements for safeguarding the drug use in children by encouraging research and development, speeding up the application and evaluation, and improving the system construction. The National Health Committee and other departments have issued three batches of pediatric drug lists for research and development by the end of 2019 [15]. With the encouraging measures, the number of clinical trial institutions qualified for conducting pediatric clinical trials in China has been significantly increased in recent years [13]. Thus, in this study, pediatric clinical trials conducted in China were analyzed to provide reference for research and development of pediatric drugs and formulation of relevant policies.

## Methods

### Data source

We conducted a cross-sectional observational study of pediatric trials registered in the Drug Trial Registration and Information Publication Platform before October 31, 2021. In 2012, the Drug Evaluation Center (CDE) of the National Medical Products Administration (NMPA) established a Drug Trial Registration and Information Publication Platform, which is a national authoritative database for clinical trials in China [16]. All drug clinical trials being conducted as registration trials (for drug marketing application and administrative approval) including phase I–IV drug trials and bioequivalence studies must be registered on the platform before enrolment of the first patient, and the NMPA is responsible for the validity and integrity of the data [17], this will be useful to increase transparency of clinical trial results [18]. The database was officially released in 2013 and retrospective registration was required for trials that conducted before 2013, but still in the new drug approval process. Publicly accessible information in the platform includes trial status, sponsor, study design, and study institutions.

### Trials screening and data extraction

Trials registered on the Drug Trial Registration and Information Publication Platform through October 31, 2021 were screened for inclusion. Clinical trials that recruited children (under 18 years old as defined in China) were included in this study. To ensure all trials conducted in children were retrieved, trials with registered information containing “prematurity”, “neonate”, “infant”, “adolescent”, “children”, “pediatrics”, “1 year” to “18 years” old, “one month” to “12 months” old were retrieved for inclusion, and trials carried out in children’s hospital and centers for disease control and prevention were all retrieved. Two authors (Lin Song and Bennian Huo) respectively screened the retrieved trials, and excluded the duplication, repeated registered trials, trials including only adults and trials that had been suspended.

Two authors (Lin Song and Bennian Huo) reviewed the full information of all the included trials and extracted the study characteristics, and any disagreements were resolved through discussion or by consulting a third author (Yao Liu). The following information of the trials, including registration number, trial status, trial phase, date of first ethical approval, province of the sponsor located, province of the principal investigator located, number of research centers, name and type, dosage form, indication of the research drug, age and target enrollment number of the subjects, whether a data monitoring committee (DMC) had been established, and whether insurance had been purchased for participants, were recorded. We defined the first ethical approval date of the trials as the date of the trials to make annual trial number statistics. The provinces of the sponsor and the principal investigator were divided into seven regions according to China’s seven geographical divisions, the north, east, south, central, northeast, northwest and southwest. For foreign sponsors, their Chinese registered province was recorded. In this study, indications of the research drugs were coded according to the International Statistic Classification of Diseases and Related Health Problems, Tenth Revision, International Classification of Diseases (ICD)-10 classification.

### Statistical analysis

Descriptive analyses were used to summarize the data, frequency (percentage) was used for qualitative data, and median (range) was used for quantitative data. A simple regression model was used to analyze the trends in the number of trials included, with *P* < 0.05 representing a statistically significant difference, and an average annual growth rate of the trials was calculated according to the average speed of growth. The year of the trial was defined by the date of the first ethical approval. All statistical analyses were performed on a personal computer with the statistical package SPSS for Windows (version 22.0).

## Results

A total of 14,907 drug clinical trials were registered on the Drug Trial Registration and Information Publication Platform as of October 31, 2021. Among the registered trials, 618 trials were retrieved and screened for inclusion, and 588 trials were included for data extraction and analysis after 17 repeated registrations and 13 suspended trials were excluded, accounting for 3.94% of the total registered number.

### General characteristics of the trials

**Fig. 1:**
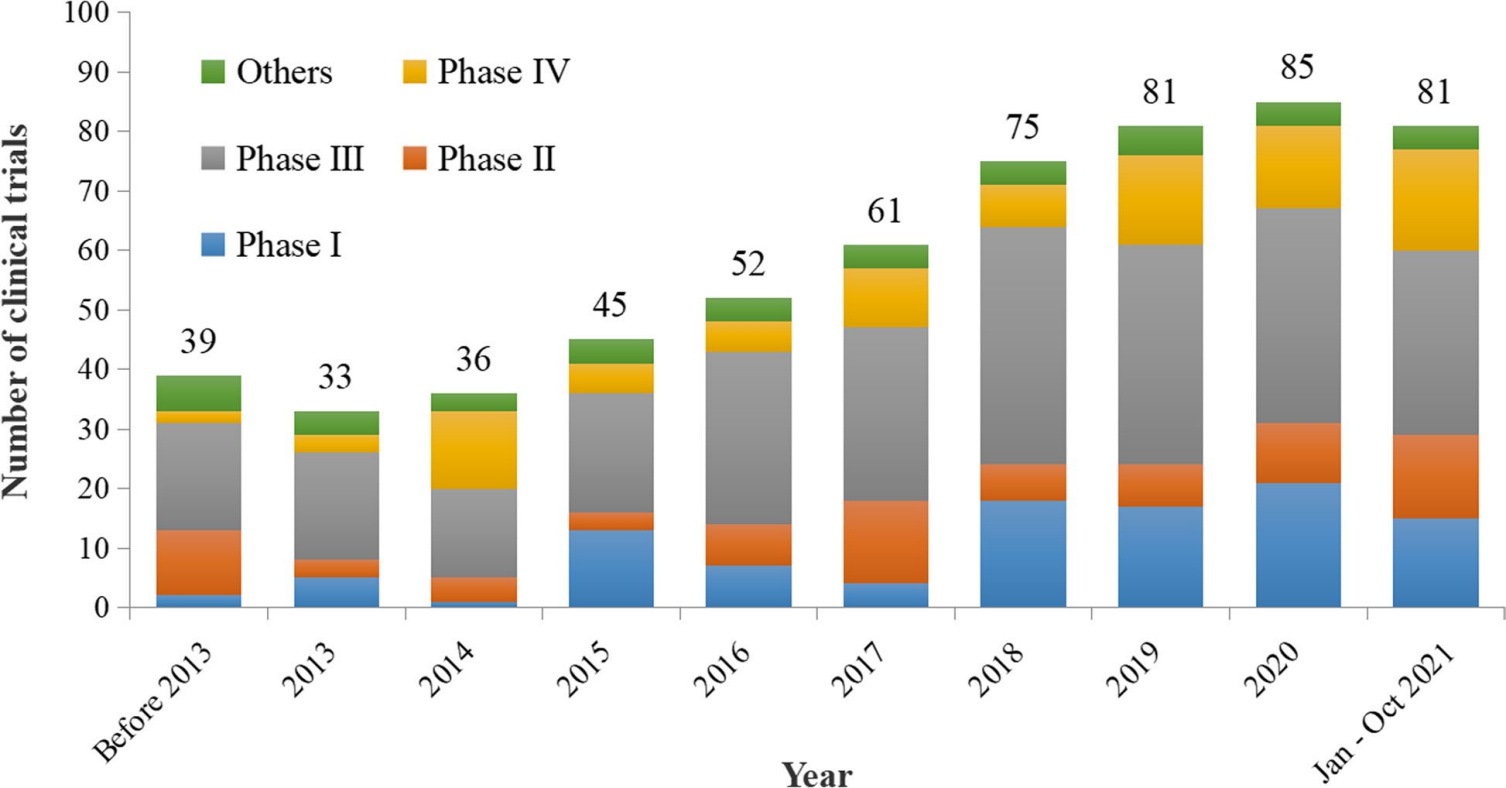
Number of pediatric clinical trials in China (the upper end of the column was marked as the total number)

Of the 588 trials, the initial registration date of the trials was from September 18, 2013 to October 29, 2021, and the first ethical approval date of the trials was from July 19, 2006 to October 9, 2021. By considering the first ethical approval date, the number and the phase of trials distributed in different years was shown in Fig. 1. A simple regression model revealed that the overall average annual growth rate of the number of trials through 2013 to 2020 was 14.47% (*P* < 0.01).

General characteristics of the trials were shown in Table 1. There were 312 trials (53.06%) with only children as subject, 127 trials (21.60%) with research drugs only for children use. 1 trial only included prematurity (gestational age 28 weeks to 33 weeks) and the research drug was caffeine citrate for apnea of prematurity. 2 trials only included neonates (< 28 days old) and the research drugs were pig lung surfactant suspension for neonatal respiratory distress syndrome and hepatitis b vaccine for neonatal hepatitis b prevention. 94 trials only included infants (< 3 years old) and the research drugs were nirsevimab injection, odevixibat capsule, maralixibat oral solution, risdiplam powder for oral solution, ibuprofen injection, timolol maleate gel, compound clove basil, other drugs with code names or vaccines (82 trials).

**Table 1:** General characteristics of 588 pediatric clinical trials in China

Items	Frequency (%)
**Trial status**	
Ongoing	372 (63.27%)
Completed	216 (36.73%)
**Trial phase**	
Phase I	103 (17.52%)
Phase II	79 (13.44%)
Phase III	273 (46.43%)
Phase IV	91 (15.48%)
Others	42 (7.14%)
Subjects^a^	
Prematurity	1 (0.17%)
Neonates	2 (0.34%)
Infants	94 (15.99%)
Toddlers and school children	113 (19.22%)
Adolescents	102 (17.35%)
Children and adults	276 (46.94%)
**Trial range**	
China	521 (88.61%)
International	67 (11.39%)
**No. of centers**	
Single-centre trial	107 (18.20%)
Multiple-centre trial	481 (81.80%)
**Target subject number** ^b^	
Phase I	100 (8, 588)
Phase II	240 (20, 1980)
Phase III	701.5 (28, 20,000)
Phase IV	600 (10, 600,000)
Others	395 (12, 16,000)
**DMC had been establish**	76 (12.93%)
**Insurance had been purchased**	241 (40.99%)

^a^Children were divided to prematurity, neonates (< 28 days old), infants (< 3 years old), toddlers and school children (< 13 years old), and adolescents (13 ~ 18 years old)

^b^Median (range) was used for target subject number

Considering the 481 multiple-center trials, the median number of centers was 5 (2 ~ 377), and the trial with 377 centers was an international multiple-center phase IV clinical trial study on compound mometasone furoate / formoterol fumarate inhalation preparation. In addition, 65 of the 76 trials for which a data safety monitoring committee had been established were multiple-center clinical trials. The median of target subject number was 320 with the range of 8 to 600,000.

### Geographical location of the trials

**Fig. 2:**
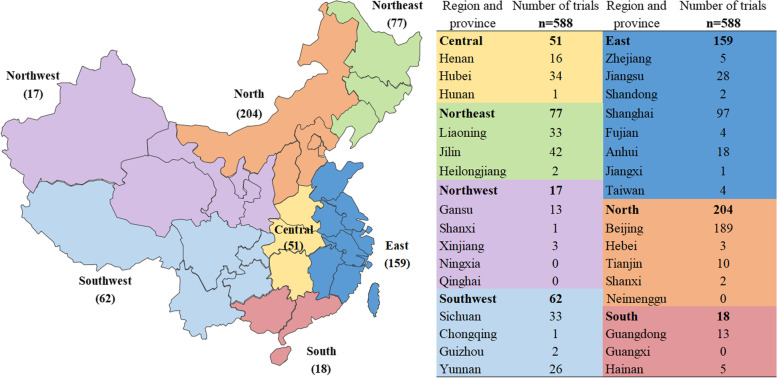
Geographical distribution of trial sponsors (the map depicted in Fig. 2 was created by our engineers from the information center using Excel 2010)

**Fig. 3:**
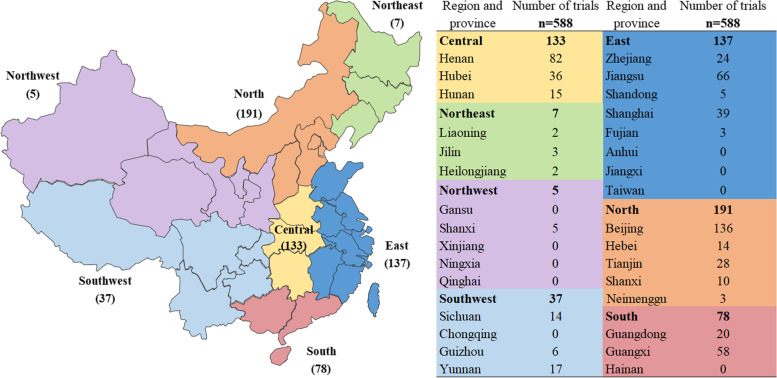
Geographical distribution of principal investigators (the map depicted in Fig. 3 was created by our engineers from the information center using Excel 2010)

The geographical distribution of the sponsors of the 588 trials was shown in Fig. 2. More than 60% of the sponsors were located in the eastern and northern China, mainly Shanghai and Beijing. The geographical distribution of the principal investigator involved in the 588 trials was shown in Fig. 3, and the trial institutions were mainly distributed in the north, east and the central China, with Beijing (mainly chemical compound drug trials), Henan and Jiangsu (both mainly vaccine trials) as the major ones.

### Distribution of the research drug types and the dosage forms

325 trials were vaccine trials, of which 215 (66.15%) were viral vaccines, and 110 (33.85%) were bacterial vaccines. The main viruses involved were rabies virus, EV71 virus, hepatitis b virus, rotavirus, mumps virus, poliovirus, influenza virus, etc. 8 rotavirus vaccines and a poliovirus vaccine were oral solutions, and all other viral vaccines were injections. The main bacteria associated vaccines were meningococcus vaccines, pneumococcus vaccines, diphtheria, tetanus and pertussis vaccines. A cholera vaccine was oral suspension, a helicobacter pylori vaccine was capsule and all other bacterial vaccines were injections.

**Fig. 4:**
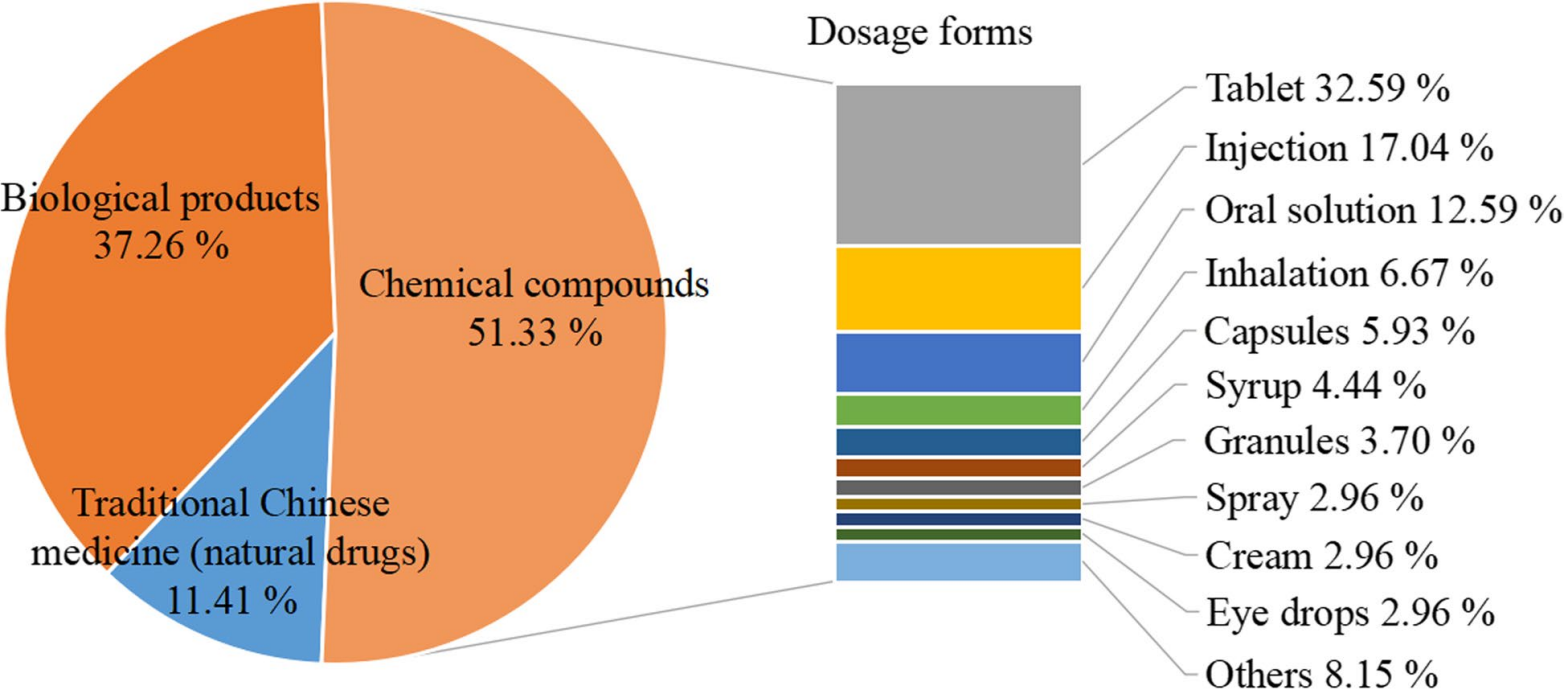
Drug types and main dosage forms of the non-vaccine research drugs (*n* = 263)

The types of the other 263 research drugs and the main dosage forms were shown in Fig. 4. There were 98 non-vaccine biological products, mainly recombinant human growth hormone, recombinant human coagulation factor, and monoclonal antibodies, and 94 (95.92%) of them were injections. Considering the dosage forms of the 135 chemical compound drugs, tablets were the main ones, followed by injections and oral solutions. 27 (90%) of the 30 traditional Chinese medicine/natural drugs were special drugs for children, and their dosage form was mainly granules (53.33%), and others were oral solution, syrup, paste, etc.

### Distribution of the indications of the research drugs

**Fig 5:**
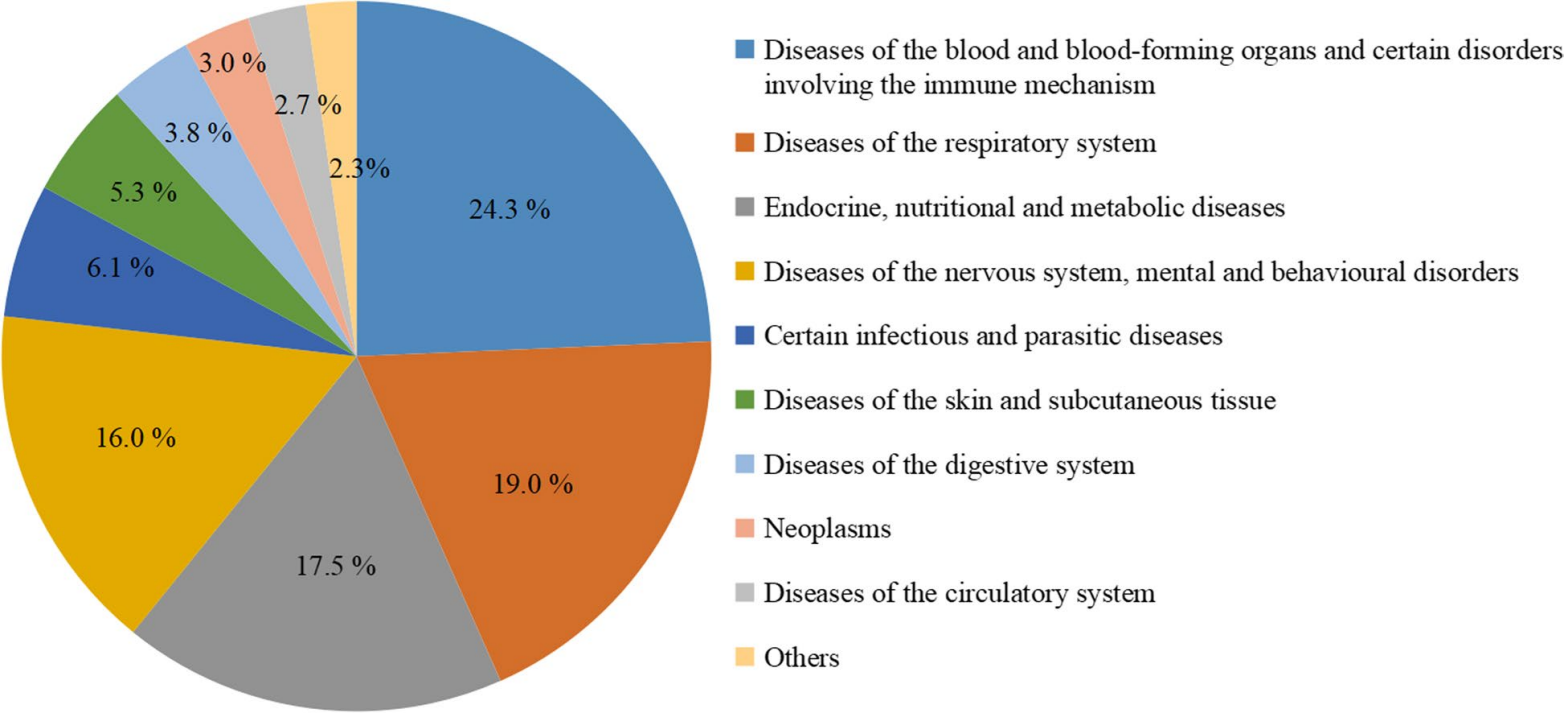
Distribution of indications of the non-vaccine research drugs (*n* = 263)

According to ICD-10, the organ system of diseases related to the indications of the 263 non-vaccine research drugs was shown in Fig. 5. Among them, 64 drugs (24.3%) were indicated for diseases of the blood and blood-forming organs and certain disorders involving the immune mechanism, followed by respiratory, endocrine and neurological diseases. There were 38 trials for rare diseases including hemophilia, mucopolysaccharidosis, fabry disease, multiple sclerosis, gaucher’s disease, spinal muscular atrophy, prader-willi syndrome.

## Discussion

The Drug Trial Registration and Information Publication Platform was started to operate in November 2012 but was not officially applied until 2013, so the pediatric clinical trials included in this study did not completely incorporate all the trials that obtained approval documents before 2012, and we only analyzed the trends in the number of trials through 2013 to 2020.

In this study, we found there were 588 registered pediatric clinical trials, and all the purpose was approval of marketing by regulators. Other pediatric clinical trials like investigator initiated clinical trials conducted in China could also be registered in the Clinical Trial. gov and the Chinese Clinical Trial Registry (ChiCTR) databases, and a previously study showed a total of 1388 pediatric clinical trials conducted in China registered in these two databases before April, 2019 [19], but in our study, only 302 trial were registered before April, 2019, indicating only about 21.8% of the pediatric clinical study results were used for drug marketing application and administrative approval before 2019 in China.

Based on the Drug Trial Registration and Information Publication Platform, we found 3.94% of the registered trials involved children, and vaccine trials accounted for more than half of the total trials (*n* = 325, 66.15%), and drug clinical trial for diseases of the blood and blood-forming organs and certain disorders involving the immune mechanism was the largest, but according to the previously study [19], 9.74% of the registered trials in Clinical Trial. gov and ChiCTR databases involved children in China, and the number of clinical trials for antineoplastic agents (*n* = 254, 18.3%), anti-infectives (*n* = 156, 11.2%) and vaccines (*n* = 154, 11.1%) was the largest, it showed that distribution of the indications of the research drugs was quite different between drug registration trials and non- registration trials. Another research based on the ClinicalTrials.gov database showed nearly 53,000 trials related to children were registered from January 2008 to December 2019, indicating less than 14.7% of the registered trials involved children all over the word [20].

According to the National Bureau of Statistics in China [21], there were 1158.4 million people over 15 years old and 253.38 million people under 15 years old in 2020. In our study, there were 563 trials that recruiting children under 15 years old, thus, there were 2.22 clinical trials that recruit children under 15 years old per million of the group; and there were 14,679 trials that including people over 15 years old in October 2021, thus, there were 12.67 clinical trials that recruit people over 15 years old per million of the group, much more than children under 15 years old group.

Due to the ontogeny of children, results of trials in adults cannot simply be applied to children. As a special and vulnerable group, although regulations and guidance for promotion clinical trials in children have been announced in different countries, performing drug trials in pediatrics is still challenging. Specialized facilities and infrastructure are essential, and shortage of pediatricians especially experienced pediatric clinical investigator is an urgent problem to be solved in China [22]. In terms of technology, improving the use of efficient databases such as electronic medical record system [23], strategies developed to increase both efficiency and safety of pediatric drug trials [24], facilities coordinated in research networks, are all needed to be considered to improve pediatric trials.

Considering the issue of greatest concern, informed consent and subject recruitment, a previous study showed the average consent rate for pediatric randomized controlled trials was 82.6% [25]. In order for clinical trial accrual to be successful, parents’ priorities and considerations must be a central focus, beginning with initial trial design, and the recommendations from the parents who participated in a clinical trial can be used to support budget allocations that ensure adequate training of study staff and improved staffing on nights and weekends [26]. Parental race/ethnicity, parental income, perception of infant’s illness severity, infant’s medicaid status, and trust in medical researchers were identified factors associated with the decision to participate in neonatal clinical trials [27].

Large, randomized controlled trials (RCTs) are essential in answering pivotal questions in child health. A previous study showed there were 247 large nonvaccine, noncluster pediatric RCTs registered in ClinicalTrials.gov before January 9, 2020, with over 1000 participants, and 40% of the trials were from high-income countries, and 49% from lower-middle-income countries, 43% of investigators in lower-income countries were from high-income countries [28]. In this study, we found there were 172 nonvaccine trials with only pediatric participants, and only 2 phase IV trials of them with over 1000 participants, and another 11 trials with over 500 participants, indicating large pediatric clinical trials need for further attention in China.

Lack of age-appropriate commercially drug products availability is a common problem in pediatric therapeutics, and tablets, effervescent tablets, capsules were considered as not age-appropriate forms. Liquid forms, powder for oral suspension, mini tablets, granules, and soluble films were considered as age-appropriate forms due to their flexibility. More than 80% of the studied drugs possess a commercial authorization in oral forms in both EMA and FDA, and around 50% of these formulations are not age-appropriate for most pediatric groups [29]. In this study, biological products were mainly injection; and nearly 50% of the chemical compound drugs were tablets and injections, and oral solution, syrup, granules account for nearly 20%, development of dosage forms suitable for children is another current challenge, especially for neonates, which have long been neglected concerning the development of oral dosage forms [30].

There were only 3 prematurity and neonates trials. Studies have shown that the incidence rate of off-label use of drugs in neonates was about 90% [31], and clinical trials in young children are facing greater ethical challenges, especially the sick neonates and prematurity, and might require new incentives or relevant policies to select the drugs that are mostly needed for research in trial design and development [32]. In addition, there are still large uneven geographical distribution of the sponsors and the research institutions, with obvious advantages in the east and the north, further narrowing regional disparities is another challenge in China.

In general, the number of pediatric clinical trials in China has continued to grow after 2019. With the revision of China’s Drug Administration Law in 2019, and with release of relevant documents to encourage research and development of medicines for children, efforts are being made to improve the lack of child-specific medicines. In addition to the suggestions mentioned above, how to push some companies to focus on developing of pediatric medicines, and how to solve the high cost and low profits of research and development of children’s medicines, formulation and implementation of relevant measures is still an issue to consider.

## Conclusion

The number of pediatric clinical trials in China has increased these years. To further promote pediatric clinical trials and motivate pediatric appropriate drug marketing application and administrative approval, conducting large pediatric clinical trials, further development of dosage forms suitable for children with special attention to neonates and prematurity, and improving uneven geographical distribution of sponsors and researchers are the current challenges. More efforts are needed to improve development and approval of pediatric drugs.

## Data Availability

The datasets used and/or analyzed during the current study are available from the corresponding author on reasonable request.

## Abbreviations

NMPA: Medical Products Administration
DMC: Data monitoring committee
ICD: International Classification of Diseases
